# Molecular Image-Based Prediction Models of Nuclear Receptor Agonists and Antagonists Using the DeepSnap-Deep Learning Approach with the Tox21 10K Library

**DOI:** 10.3390/molecules25122764

**Published:** 2020-06-15

**Authors:** Yasunari Matsuzaka, Yoshihiro Uesawa

**Affiliations:** Department of Medical Molecular Informatics, Meiji Pharmaceutical University, Tokyo 204-8588, Japan; matsuzys@my-pharm.ac.jp

**Keywords:** chemical structure, DeepSnap, deep learning, nuclear receptor, QSAR, Tox21 10K library

## Abstract

The interaction of nuclear receptors (NRs) with chemical compounds can cause dysregulation of endocrine signaling pathways, leading to adverse health outcomes due to the disruption of natural hormones. Thus, identifying possible ligands of NRs is a crucial task for understanding the adverse outcome pathway (AOP) for human toxicity as well as the development of novel drugs. However, the experimental assessment of novel ligands remains expensive and time-consuming. Therefore, an in silico approach with a wide range of applications instead of experimental examination is highly desirable. The recently developed novel molecular image-based deep learning (DL) method, DeepSnap-DL, can produce multiple snapshots from three-dimensional (3D) chemical structures and has achieved high performance in the prediction of chemicals for toxicological evaluation. In this study, we used DeepSnap-DL to construct prediction models of 35 agonist and antagonist allosteric modulators of NRs for chemicals derived from the Tox21 10K library. We demonstrate the high performance of DeepSnap-DL in constructing prediction models. These findings may aid in interpreting the key molecular events of toxicity and support the development of new fields of machine learning to identify environmental chemicals with the potential to interact with NR signaling pathways.

## 1. Introduction

Many chemical substances have potential harmful effects, causing the perturbation of endocrine homeostasis by interfering with various nuclear receptors (NRs) of hormones [1,2,3,4,5]. In the disruption of hormone pathways, structurally diverse groups of chemicals are known to interact primarily with ligand–NR bindings, which have the ability to substitute for natural ligands, ultimately resulting in proliferative, reproductive, and metabolic disorders [6,7,8,9,10,11,12]. NRs are a superfamily of ligand-dependent transcriptional factors containing n N-terminal transactivation domain, a flexible hinge region, and a C-terminal ligand-binding domain (LBD) [6,8,13]. NRs are classified mainly into two types according to their subcellular distribution in the absence of a ligand and their mechanisms: Type I steroid receptors, including the estrogen receptor (ER), androgen receptor (AR), progesterone receptor (PR), and glucocorticoid receptor (GR); and Type II nonsteroid receptors, including the thyroid receptor (TR alpha and beta), retinoic acid receptor (RAR alpha, beta, and gamma), retinoid X receptor (RXR), vitamin D receptor (VDR), peroxisome proliferator-activated receptor (PPAR alpha, beta, and gamma), liver X receptor (LXR), farnesoid X receptor (FXR), and pregane X receptor (PXR), [6,14,15]. In the absence of a ligand, the type I NR forms inactive complexes with chaperone proteins in the cytoplasm, whereas type II NR, regardless of the ligand-binding status, is located in the nucleus and binds to the DNA response elements of its target genes along with corepressors [6,14,16]. For these types of NRs, a number of allosteric modulators have been identified that can act as either agonist or antagonist by occupying the active pocket of the NR and blocking the recruitment of coactivators or corepressors to the transcriptional complex [11,17,18,19,20].

The perturbation of the NR signaling pathway due to the action of agonists or antagonists of chemical compounds is associated with various adverse health outcomes [19,21]. Although chemical hazard assessments have traditionally relied upon toxicity data from animal bioassays and epidemiological studies, there are some drawbacks to this testing method, such as high cost, lengthy test durations, and ethical concerns [5,22,23,24,25,26,27]. To resolve these issues, the in vitro high-throughput screening (HTS) assay has been developed as an alternative approach and improved by the Toxicity Forecaster (ToxCast^TM^) program run by the U.S. Environmental Protection Agency (EPA) [5,28,29,30] and The Toxicology in the 21st Century program (Tox21), an interagency federal collaboration launched by the consortium of the EPA, the U.S. Food and Drug Administration (FDA), the National Institutes of Health (NIH), and the National Toxicology Program (NTP) [5,31]. However, the HTS assay is not sufficient to screen all classes of chemicals, such as those still in molecular development and optimization phase, and thus cannot provide an accurate evaluation of the potential toxicity of chemicals in humans and the environment [5,32].

Recent technological advances have focused on in silico approaches, such as quantitative structure–activity relationship (QSAR), based on the assumption that similar structures are associated with similar biological activities, taking advantage of their ability to accurately predict the toxicologically discrete values of the chemical or biological properties of molecules [5,33,34,35,36,37]. However, the QSAR approach has the following disadvantages: (i) required skills and knowledge for feature extraction and selection, (ii) paucity of model interpretability, and (iii) low prediction performance due to the dependence on the choice of molecular descriptors and the prediction modeling algorithms [36,38,39,40]. To address these issues, a novel deep learning (DL)-based QSAR method, called DeepSnap-DL [41], was developed using molecular image files generated from the steric conformation of three-dimensional (3D) chemical structures, leveraging the increasing evidence of successful classification by convolutional neural networks (CNNs) through DL in toxicological fields [40,42,43]. This method has the following advantages. First, the feature(s) in the molecular images can be automatically extracted by CNNs. Second, high prediction performance can be expected as more detailed information of the chemical structure can be captured from different viewing directions along the x-, y-, and z-axes [41,44,45,46,47]. Third, determination and visualization of the conformer that is docked in the LBD of the receptor protein may reveal the critical conformation of the chemicals and domain of the receptor protein related to the adverse outcome.

In this study, using the DeepSnap-DL method, prediction models of 35 agonists and antagonists of NRs were constructed by 3D molecular structure representations using information of chemical compounds from the Tox21 10K library. The results obtained by the DeepSnap-DL method outperformed those of the methods that won the Tox21 data challenge. Therefore, our approach can be practically applied to build prediction models using a CNN for a large number of chemicals to determine their potential toxicity.

## 2. Results and Discussion

**Figure 1:**
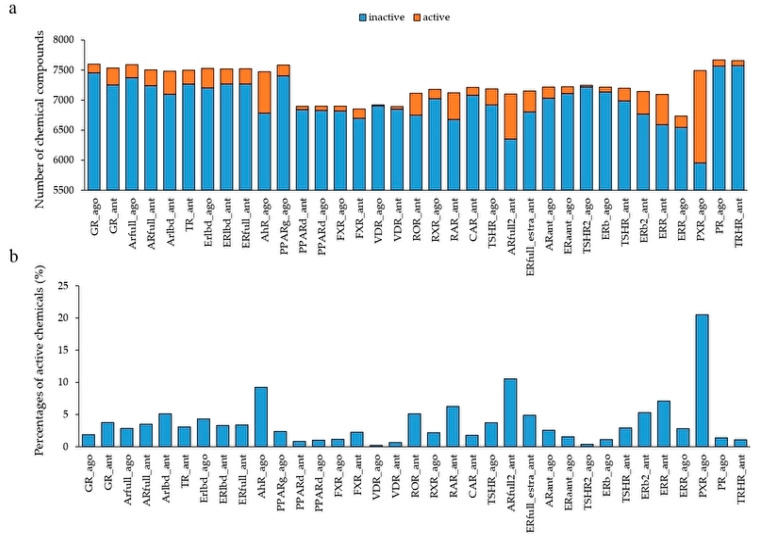
Activity distribution of the Tox21 10K library against 35 NR agonists and antagonists used in the DeepSnap-deep learning (DL) approach. (**a**) Number of chemical compounds used in the modeling by the DeepSnap-DL, where orange and blue indicate active and inactive chemicals, respectively. (**b**) Percentage of active chemicals against total chemicals.

To build the prediction models of the agonists and antagonists of NRs, we downloaded the information of 35 NRs for the chemical structures and their activity scores from the Tox21 10K library. The mean number of chemicals was 7262 ± 267, and the highest and lowest numbers of the chemicals were respectively 7671 (progesterone receptor agonist: PR_ago, AID: 1347036), and 6735 (estrogen-related receptor agonist: ERR_ago, AID: 1259404) (Figure 1). Furthermore, we classified the datasets of these chemical compounds into two groups based on their activity scores—active chemicals were those with an activity score ≥ 40 and inactive chemicals had an activity score < 40. The mean number of active chemicals in the total chemicals was 0.0372 ± 0.0376, and the highest and lowest numbers of active chemicals were, respectively, 0.2052 (pregane X receptor agonist: PXR_ago, AID: 1347033) and 0.0022 (vitamin D receptor agonist: VDR_ago, AID: 743241) (Figure 1). These results indicate that the datasets are highly class imbalanced.

**Figure 2:**
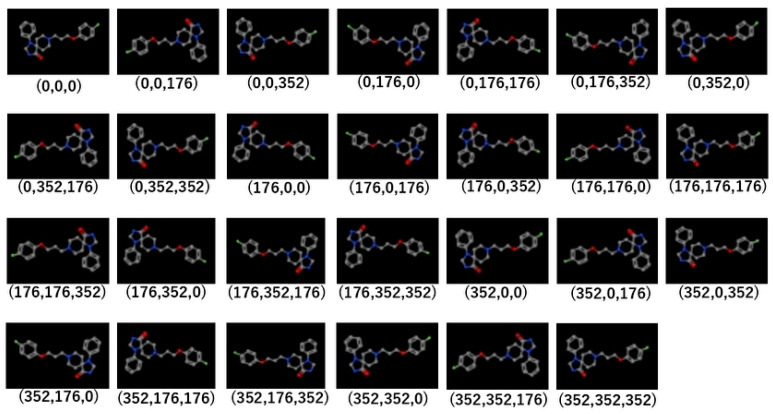
Representative molecular images used in the DeepSnap-DL method. The parentheses below each image indicates the angles of depiction in DeepSnap.

Next, the datasets were divided into Tra:Val:Test groups with a 4:4:1 ratio. The mean numbers of active and inactive chemicals were, respectively, 120.9 ± 124.1 and 3107.1 ± 153.1 in Tra, 120.8 ± 124.3 and 3106.8 ± 153.3 in Val, and 30.1 ± 30.9 and 271.8 ± 279.2 in Test (Table S1, Supplementary Materials). In addition, the highest and lowest numbers of the active chemicals were, respectively, 683 and 2 in Tra, 684 and 6 in Val, and 170 and 2 in Test (Table S1). The molecular images derived from the 3D chemical structures were generated using the DeepSnap approach at different angles along the x-, y-, and z-axes, i.e., (176°, 176°, 176°). A total of 27 images for one chemical compound was captured (Figure 2, Figure S1).

**Figure 3:**
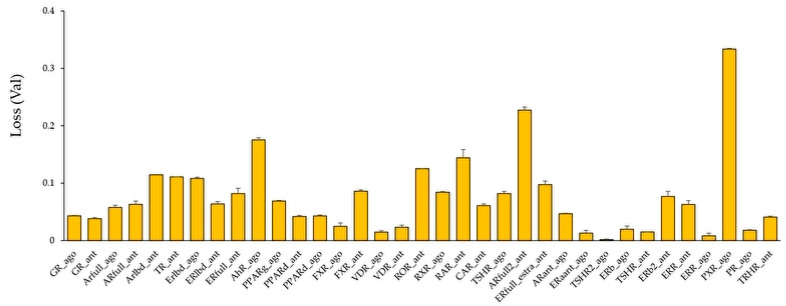
Average Loss values in the validation (Val) dataset in models of 35 NR agonists and antagonists constructed by DeepSnap-DL. *n* = 2. Each bar indicates average of Loss (Val) ± standard error.

**Figure 4:**
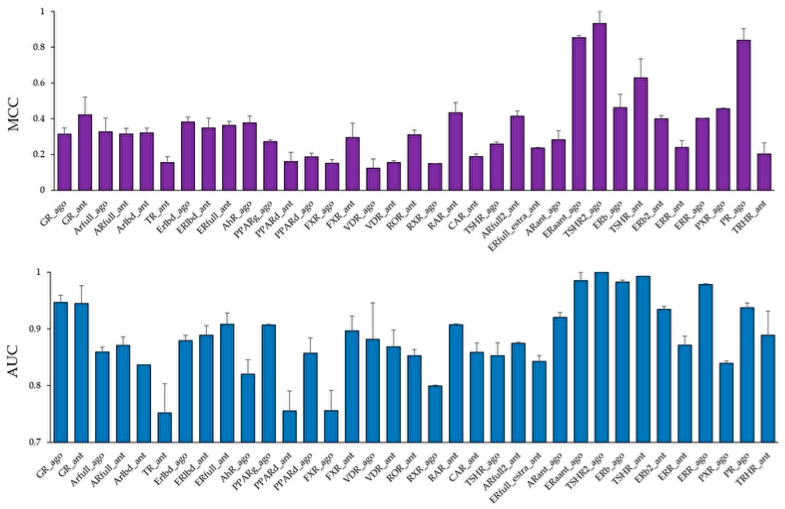
Average Matthews correlation coefficient (MCC) (**top**) and area under the curve (AUC) (**bottom**) values in the Test dataset in the models of 35 NR agonists and antagonists constructed by DeepSnap-DL. *n* = 2. Each bar indicates average MCC and AUC ± standard error.

**Figure 5:**
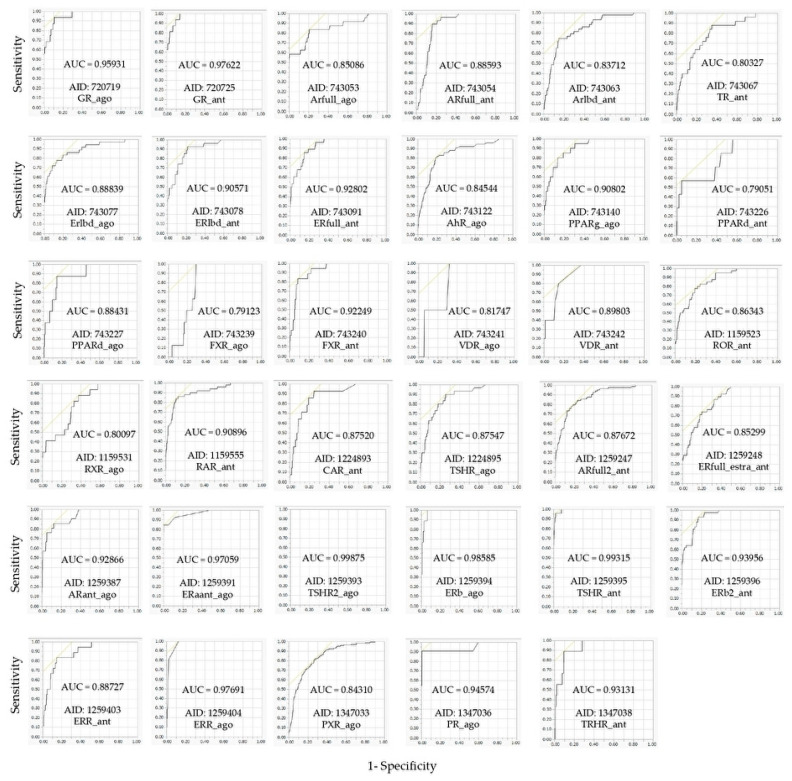
Representative area under the curve of receiver operating characteristic curve (ROC_AUC) in the models of 35 NR agonists and antagonists constructed by DeepSnap-DL.

Using these molecular images as input data into the DL, the prediction models of 35 NR agonists and antagonists were constructed using Tra, and validated with Val. The values of mean Loss (Val) and Acc (Val) were 0.0748 ± 0.0035 and 97.56 ± 0.09, respectively (Figure 3, Figure S4a, Supplementary Materials). In addition, the highest prediction performance on the Val dataset was observed in the thyroid-stimulating hormone receptor agonist (TSHR2_ago, AID: 1259393), for which the mean Loss (Val) and Acc (Val) were 0.0017 ± 0.0008 and 99.93 ± 0.02, respectively (Figure 3, Figure S4a). The prediction performance of these models was evaluated using Test based on five metrics, namely AUC, BAC, F, Acc (Test), and MCC. The results showed that the mean AUC, BAC, F, Acc (Test), and MCC were 0.8842 ± 0.0165, 0.8471 ± 0.0168, 0.3085 ± 0.0411, 82.73 ± 3.92, and 0.3536 ± 0.0377, respectively (Figure 4 and Figure 5, Figure S4a,b). In addition, the highest prediction performance on Test was observed in the thyroid-stimulating hormone receptor agonist (TSHR2_ago, AID: 1259393), with the mean AUC, BAC, F, Acc (Test), and MCC being 0.9994 ± 0.0006, 0.9997 ± 0.0003, 0.9286 ± 0.0714, 99.94 ± 0.06, and 0.9327 ± 0.0673, respectively (Figure 4 and Figure 5, Figure S4a,b).

The Tox21 Data Challenge 2014 was designed to understand the interference of the chemical compounds derived from the Tox21 10K compound library in the biological pathway via crowdsourced data analysis by independent researchers. It used data generated from seven NR signaling pathway assays to construct prediction models for QSARs [48]. The BAC values of the three models constructed by the proposed DeepSnap-DL were 0.8361, 0.8204, and 0.8494, respectively, outperforming the Data Challenge models where the BACs of three models, namely AID:743053 (Arfull_ago), AID:743077 (Erlbd_ago), and AID:743140 (PPARg_ago), were 0.6500, 0.7147, and 0.7852, respectively. However, the best prediction model of AID:743122 (AhR_ago) had a BAC value of 0.8528 in the Data Challenge, whose BAC outperformed that in the DeepSnap-DL method (0.7785). Up to now, conflicting observations have been reported regarding whether DL performs better than conventional shallow machine learning (ML) methods, such as random forest, support vector machine, and gradient boosting decision tree [40,43,49,50,51,52,53]. Although some reports suggest that DL outperforms conventional ML methods owing to various improvements, the performance of DL in terms of QSAR may be affected by many factors, such as molecular descriptors, assay targets, chemical space, hyper-parameter optimization, DL architectures, input data size, and quality [40].

Furthermore, the DeepSnap-DL approach has the black box problem, that is, it lacks explainability and interpretability of the prediction models because the convolutional area on the image picture by CNN is not defined. This issue has been extensively studied, especially in the field of image recognition. These studies try to resolve the issue by calculating the gradient of the input image with respect to the output label and highlighting the target pixel as a recognition target when a slight change in a specific input pixel causes a large change in the output label. However, a simple calculation of the gradient generates a noisy highlight, so some improved methods have been proposed for sharpening [54,55,56,57,58,59]. In addition, in the DeepSnap-DL approach, the performance improves as data size increases, and performance deterioration is observed with insufficient data size or the presence of noise. However, simply increasing the sample size causes problems such as overfitting and increased calculation costs. To resolve the issues of the DeepSnap-DL approach, critical factors include specifying the image area and type required for effective feature extraction to reduce the input data volume, and clarification of the functional relationship of chemical substances with biological activity in vivo. Future applications may include screening of target molecules in specific pathological reactions.

**Figure 6:**
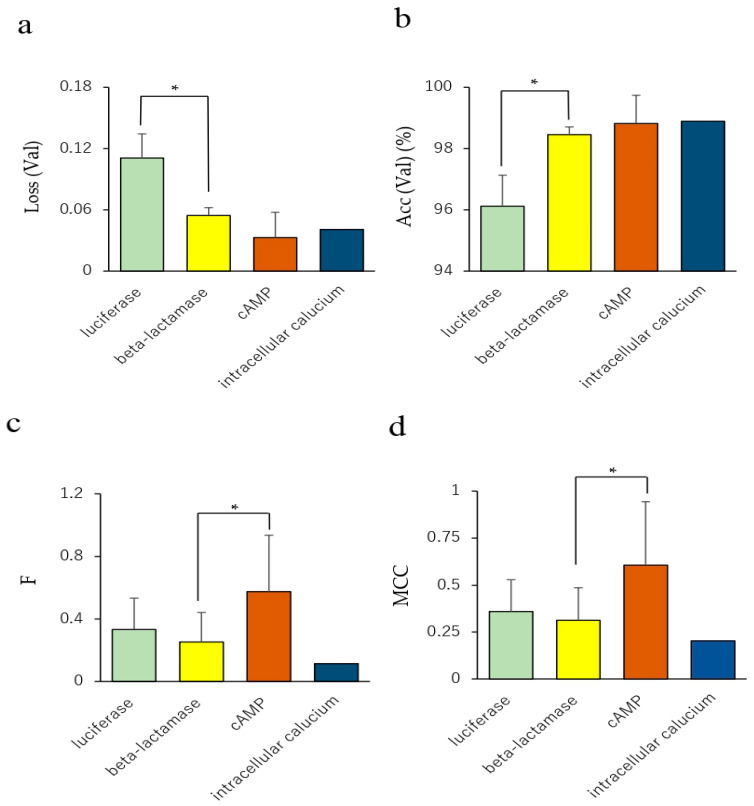
Comparison of prediction performance among four in vitro assays. (**a**) Loss in the Val dataset, (**b**) accuracy in the Val dataset, (**c**) accuracy in the Test dataset. *n* = 14, 17, 3, and 1 for luciferase, beta-lactamase, cAMP, and intracellular calcium assays, respectively. Each bar indicates the average of the performance metric of the four in vitro assays with standard error. * *p* < 0.05 by Tukey–Kramer’s honestly significant difference test.

To investigate whether the in vitro bioassays for agonist and antagonist mode in the Tox21 program affect the prediction performance of NRs, we compared prediction performances among four in vitro assays, namely, luciferase, beta-lactamase, cAMP, and intracellular calcium assays, using the results of 35 NR agonist and antagonist prediction models. In the Val dataset, the loss and accuracy values in the luciferase assay were significantly higher and lower, respectively, compared with that of the beta-lactamase assay (Figure 6a,b, *p* < 0.05 for both Loss (Val) and Acc (Val)).

In addition, F and MCC in Test of the cAMP assay significantly increased compared with those of the beta-lactamase assay (Figure 6c,d, *p* < 0.05 for both F-measure and MCC). The BAC value in the Test dataset of the cAMP assay showed a moderate increase compared with that of the beta-lactamase assay (Figure S5c, Supplementary Materials, *p* < 0.09). These results indicate that the prediction performance of the NR agonists and antagonists in the Tox21 10K library may be affected by the choice of the in vitro assay method. There are several conflicting reports regarding the in vitro receptor-mediated activity. Chemicals such as bisphenol A (BSA) and its halogenated analogs (tetrabromo-BSA and tetrachloro-BSA) show weak TR antagonist activity but have a potential agonist-like effect at lower concentrations [60,61]. Thus, competitive agonists and antagonists of the steroids have long been known [62,63,64]. Among them, ligands exhibiting agonist and antagonist activity, called selective steroid receptor modulators (SSRMs), are known to show specificity on tissue or cell type [62,65,66,67,68,69]. In addition, a competitive antagonist, known as the passive antagonist, hinders the binding but induces the inactive state of NRs by modifying interaction with their corepressor and interfering with their nuclear translocation or DNA binding at saturated concentrations [62,70]. These reports suggest that the ligand of the steroid NRs can serve not only as competitive agonists and antagonists that affect binding to the NRs, but also as a unique allosteric modulator for subsequent molecular interactions. Therefore, classification of the chemicals in the Tox21 10K library may require more detailed insights of the molecular mechanisms of the NRs with chemical compounds and the conditions of in vitro bioassays.

## 3. Conclusions

In this study, we built prediction models of 35 NR agonists and antagonists using the DeepSnap-DL approach with information of the chemical structure and activity from the Tox21 10K library. Three prediction models outperformed the best performing models in the Tox21 Data Challenge 2014. These results suggest that the 3D chemical structure representation in the DeepSnap-DL approach may be useful for molecular image-based QSAR analysis, and the improvements to the DeepSnap-DL method may aid in achieving high-performing prediction models.

## 4. Materials and Methods

### 4.1. Data

In this study, the original datasets related to chemical structures and the corresponding agonist and antagonist scores were downloaded as reported previously [44,45,46,47], in the simplified molecular input line entry system (SMILES) format from the PubChem database. We used a keyword of the database search, namely “Tox21 bioassays”, and selected bioassays of the 35 from the NR signaling pathway for the identification of agonists/antagonists (Table 1). These bioassay data consisted of quantitative HTS (qHTS) data derived from two cell-based reporter gene assays, including beta-lactamase or luciferase reporter genes. The activity of these reporter genes is controlled by the binding of transcriptional factors induced or suppressed by an agonist/antagonist with response elements (REs) for ARs, ER-alpha, ER-beta, estrogen-related receptors (ERR), FXR, PPAR−gamma, PRs, retinoid-related orphan receptor gamma (ROR−gamma), RXR−alpha, RARs, GRs, TRs, thyroid-stimulating hormone receptors (TSHRs), aryl hydrocarbon receptors (AhRs), VDRs, constitutive androstane receptors (CARs), and PXRs. These receptors are stably integrated into cell lines, including human embryonic kidney 293 cells(HEK293 (AR, ER−alpha, ER−beta, ERR, and TSHR), HEK293H (PPAR−gamma, PPAR−delta, and HEK293T (ER−beta, FXR, PR, RXR−alpha, and VDR)), human breast cancer cells (MDA−MB (AR)), ovarian carcinoma cells (BG1 (ER−alpha)), Chinese hamster ovary cells CHO (ROR−gamma)), human cervical cancer cells (HeLa (GR)), rat pituitary tumor cells (GH3 (TR)), human hepatocellular carcinoma cells (HepG2 (AhR, CAR, PXR)), and C3H mouse embryo cells (C3RL4 (RXR−alpha)). Then, we can measure the ability to induce or inhibit RE-dependent transcription.

The chemicals were derived from the Tox21 10K library, which contains approximately 8900 unique compounds gathered from commercial sources, such as pesticides, industrial and environmental chemicals, natural dietary supplement products, food additives, and drugs, by the NTP, the National Center for Advancing Translational Sciences (NCATS), and the EPA (Table 1) [71,72,73,74,75,76,77,78,79,80,81,82]. These compounds were dissolved in dimethyl sulfoxide (DMSO) as stock solutions, and compound plates with the different concentrations were prepared in the 1536-well plate format [71,72,73,80,83]. These cell lines of beta-lactamase reporter gene assay constitutively co-express a fusion protein comprised of the LBDs of the human NRs coupled to the DNA-binding domain (DBD) of the yeast transcription factor GAL4 [72,73,75,80]. When activated, these fusion proteins stimulate beta-lactamase reporter gene expression.

The cells were dispensed at 1500 to 5000 cells/5 (for antagonist mode) or 6 (for agonist mode) microL/well in 1536-well black wall/clear bottom plates [72,73,75,78,79,80]. After the cells were incubated at 37 °C for 5 to 6 h depending on the particular NR cell line to allow for cell attachment, 23 nL of the compounds at different concentrations were transferred to the assay plates. For the antagonist mode assay, the known agonist for each NR was added into the assay plates. For the agonist and antagonist mode assays, positive control compounds were dispensed into each other’s wells on the plates (Table 1) [72,73,75,78,79,80]. The plates were incubated for 16 to 18 h at 37 °C depending on the particular NR cell line. Then, a LiveBLAzer^TM^ B/G FRET substrate (Invitrogen, Carlsbad, CA, USA) detection mix was added, and the plates were incubated at room temperature for 1.5 to 2 h. The fluorescence intensity (405 nm excitation, 460 and 530 nm emission) was measured using an Envision plate reader (PerkinElmer, Shelton, CT, USA). Data were expressed as the ratio of 460/530 nm emission values. To measure the luciferase reporter gene activity, 4 microL of ONE-Glo^TM^ Luciferase Assay reagent (Promega, Madison, WI, USA) were added to each plate, and the luminescence intensity was quantified by a ViewLux plate reader (PerkinElmer) after 30 min of incubation at room temperature. Data were expressed as relative luminescence units.

### 4.2. qHTS Data Analysis

The Tox21 10k library can be grouped into clusters with similar activity that share similar annotated models of action according to PubChem activity scores. In the qHTS of the Tox21 program, to identify the chemical compounds in both potential agonist and antagonist modes, the PubChem activity scores were determined from 0% to 100% by normalizing each titration point relative to the positive control compound (agonist mode: 100%, antagonist mode: 0%) and DMSO-only wells (agonist mode: 0%, antagonist mode: -100%) according to the following equation: % Activity = [(Vcompound − Vdmso)/(Vpos − Vdmso)] × 100, where Vcompound, Vdmso, and Vpos denote the compound well values, the median values of the DMSO-only wells, and the median value of the positive control well, respectively.

The datasets were then corrected using compound-free control plates, i.e., DMSO-only plates, at the beginning and end of the compound plate measurement [72,73,75,78,79,80]. The half maximum inhibition values (IC_50_) and the maximum response values for each compound were calculated by fitting the concentration–response curves of each compound to a four-parameter Hill equation [84,85].

The PubCem activity scores of the agonists and antagonists were grouped into three classes, namely (1) 0, (2) 1–39, and (3) 40–100, which represent inactive, inconclusive, and active compounds, respectively. In this study, compounds with activity scores of 40–100 or 0–39 were defined as active or inactive, respectively. The dataset includes some similar chemical compounds, but with different activity scores for different ID (identification) numbers due to the presence of possible stereoisomers or salts. Therefore, chemical compounds with indefinite activity criteria, nonorganic compounds, and/or inaccurate SMILES were eliminated.

### 4.3. DeepSnap

We then applied a 3D conformational import from the SMILES format using molecular operating environment (MOE) 2018 software (MOLSIS Inc., Tokyo, Japan) to generate the chemical database. Here, the neutralization of the protonation state and the coordinating washed species were used by the external program, CORINA classic software (Figure S1, Supplementary Materials) [86]. The resulting 3D structures were then saved in an SDF file format. Using the SDF files prepared by the MOE application, the 3D chemical structures were depicted as 3D ball-and-stick models with different colors corresponding to different atoms by Jmol, an open-source Java viewer software (version number, manufacturer, city, state abbreviation, country) for 3D molecular modeling of chemical structures [44,45,46,47]. These 3D chemical structures produce different images depending on the direction. The 3D chemical models were captured automatically as snapshots with user-defined angle increments with respect to the x-, y-, and z-axes. In this study, one angle increment was used, i.e., (176°, 176°, 176°). Other parameters for the DeepSnap depiction process were set based on previous studies as follows: image pixel size: 256 × 256; molecule number per SDF file to split into: 100; zoom factor (%): 100; atom size for van der Waals radius (%): 23; bond radius (mÅ): 14.5; minimum bond distance: 0.4; and bond tolerance: 0.8 [44,45,46,47]. The snapshots saved as 256 × 256 pixel resolution PNG files (RGB) were divided into three types of datasets: training (Tra), validation (Val), and test (Test) (Figure S1, Figure 2).

### 4.4. Preparation of Dataset

Three groups of datasets were prepared by dividing the data into Tra, Val, and Test groups. The data were first split into 11 groups, and the two dataset groups (4:4:1_01 and 4:4:1_02) were then built in accordance with the ratio of Tra:Val:Test = 4:4:1. A prediction model was created using the Tra and Val datasets. Then, the prediction performance was evaluated using the Test dataset (4:4:1_01) (Figure S2, Supplementary Materials). For a subsequent analysis, the remaining Test dataset was selected from the group not used in the first analysis. The model was then built, and its probability calculation was examined in the same manner (4:4:1_02). Finally, two tests were performed and the average was calculated (Figure S2).

### 4.5. Deep Learning

All the two-dimensional (2D) PNG images produced by DeepSnap were resized by utilizing the NVIDIA DL GPU Training System (DIGITS) version 4.0.0 software (NVIDIA, Santa Clara, CA, USA), on four-GPU systems, Tesla-V100-PCIE (31.7 GB), with a resolution of 256 × 256 pixels as input data, as previously reported [44,45,46,47]. The prediction model was pre-trained as transfer learning [44,45,46,47] by the ImageNet Large Scale Visual Recognition Challenge (ILSVRC) 2012 dataset [87], which includes 1000 classes, such as animal (40%), device (12%), container (9%), consumer goods (6%), and equipment (4%). The ILSVRC 2012 dataset was divided as 1.2 million Tra, 50,000 Val, and 1 million Test datasets extracted from ImageNet [88]. To rapidly train and fine-tune the highly accurate CNNs using the input Tra and Val datasets based on the image classification and building the pre-trained prediction model, we used a pre-trained open-source DL model, Caffe, and the open-source software on the CentOS Linux distribution 7.3.1611. In this study, the deep CNN architecture was GoogLeNet, which is a complex network inspired by LeNet and implemented with a novel module called “Inception”, which facilitates batch normalization, image distortions, and RMSprop; concatenates different filter sizes and dimensions into a single new filter; and introduces sparsity and multiscale information in one block (Figure S3, Supplementary Materials). The network is a 22-layer deep CNN, comprising two convolutional layers, two types of pooling layers (four max pools and one avg pool), and nine Inception modules, each module having six convolution layers and one pooling layer, with 4 million parameters (Figure S3) [89,90,91].

In the DeepSnap-DL method, the prediction models were constructed by training datasets using 30 epochs with 1 snapshot interval in each epoch, 1 validation interval in each epoch, 1 random seed, a stochastic gradient descent-type solver, a learning rate of 0.006, and a batch size of 108 in DL. Among the epochs, the lowest Loss value in the Val dataset (Loss (Val)), which is the error rate between the results obtained from the validation data and the corresponding labeled dataset, was selected for subsequent examination of prediction using the Test dataset.

### 4.6. Evaluation of the Predictive Model

Through two tests conducted on the Test datasets for the experiments, with Tra:Val:Test = 4:4:1 in the DL prediction model, we analyzed the probability of the prediction results with the lowest minimum Loss (Val) value among 30 examined epochs. We calculated the probabilities for each image of one molecule captured at different angles with respect to the x-, y-, and z-axes using DeepSnap-DL. The medians of each of these predicted values were used as the representative values for target molecules as previously reported [44,45,46,47]. The performance of each model in predicting the NR agonists and antagonists was evaluated in terms of the following metrics: area under the curve of receiver operating characteristic curve (ROC_AUC); balanced accuracy (BAC); accuracy (Acc), which is the percentage of correct answers based on the results obtained from the validation dataset and the corresponding labeled dataset; F-measure; and Matthews correlation coefficient (MCC) calculated using JMP Pro 14, which is a statistical discovery software (SAS Institute Inc., Cary, NC, USA), as previously reported [44,45,46,47]. These performance metrics are defined as follows:

BAC = (sensitivity + specificity)/2, where

Sensitivity = ΣTPs / (ΣTPs + ΣFNs),

Specificity = ΣTNs / (ΣTNs + ΣFPs),

Accuracy = (TP + TN) / (TP + FP + TN + FN),

F-measure = 2 × Recall × Precision / (Recall + Precision), where

Precision = TP / (TP + FP),

Recall = TP / (TP + FN),

$$\mathrm{MCC}=(\mathrm{TP}\times \mathrm{TN}-\mathrm{FP}\times \mathrm{FN})/\sqrt{\left(\mathrm{TP}+\mathrm{FP}\right)\times \left(\mathrm{TP}+\mathrm{FN}\right)\times \left(\mathrm{TN}+\mathrm{FP}\right)\times \left(\mathrm{TN}+\mathrm{FN}\right)},$$

where TP, FN, TN, and FP denote true positive, false negative, true negative, and false positive, respectively. To determine the optimal cutoff point for the definition of TP, FN, TN, and FP, the method of maximizing sensitivity (1–specificity), which is called the Youden index [92,93], was adopted using JMP Pro software. The index has a value ranging from 0 to 1, where 1 represents maximum effectiveness and 0 represents minimum effectiveness.

### 4.7. Statistical Analysis

Differences in prediction performance of in vitro assays in terms of loss (Val), Acc (Val), and Acc (Test), were analyzed by Tukey–Kramer’s honestly significant difference test with JMP Pro 14 [94]. Results with *p* < 0.05 were considered statistically significant.

## Figures and Tables

**Table 1:** Nuclear receptors (NRs) and their bioassays used in this study.

PubChem AID	Model Names	NRs	Activity	Reporter Gene Assay	Cell Lines	Agonist/Antagonist	Positive Control
720719	GR_ago	glucocorticoid receptor	agonist	beta-lactamase	HeLa		Dexamethasone
720725	GR_ant	glucocorticoid receptor	antagonist	beta-lactamase	HeLa	Dexamethasone	Mifeprostone
743053	Arfull_ago	androgen receptor	agonist	beta-lactamase	HEK293		R1881
743054	ARfull_ant	androgen receptor	antagonist	luciferase	MDA-MB	R1881	Nilutamide
743063	Arlbd_ant	androgen receptor	antagonist	beta-lactamase	HEK293	R1881	Cyproterone acetate
743067	TR_ant	thyroid receptor	antagonist	luciferase	GH3	T3	NA
743077	Erlbd_ago	estrogen receptor alpha	agonist	beta-lactamase	HEK293		17beta-estradiol
743078	ERlbd_ant	estrogen receptor alpha	antagonist	beta-lactamase	HEK293	17beta-estradiol	4-Hydroxy tamoxifen
743091	ERfull_ant	estrogen receptor alpha	antagonist	luciferase	BG1	17beta-estradiol	4-Hydroxy tamoxifen
743122	AhR_ago	aryl hydrocarbon receptor	agonist	luciferase	HepG2		Omeprazole
743140	PPARg_ago	peroxisome proliferator-activated receptor gamma	agonist	beta-lactamase	HEK293H		Rosiglitazone
743226	PPARd_ant	peroxisome proliferator-activated receptor delta	antagonist	beta-lactamase	HEK293H	L-165041	MK886
743227	PPARd_ago	peroxisome proliferator-activated receptor delta	agonist	beta-lactamase	HEK293H		L-165041
743239	FXR_ago	farnesoid-X-receptor	agonist	beta-lactamase	HEK293T		Chenodeoxycholic acid
743240	FXR_ant	farnesoid-X-receptor	antagonist	beta-lactamase	HEK293T	Chenodeoxycholic acid	Guggulsterone
743241	VDR_ago	vitamin D receptor	agonist	beta-lactamase	HEK293T		1alpha, 25-Dihydroxy Vitamin D3
743242	VDR_ant	vitamin D receptor	antagonist	beta-lactamase	HEK293T	1alpha, 25-Dihydroxy Vitamin D3	NA
1159523	ROR_ant	retinoid-related orphan receptor gamma	antagonist	luciferase	CHO	Doxycycline Hyclate	TO-901317
1159531	RXR_ago	retinoid X nuclear receptor alpha	agonist	beta-lactamase	HEK293T		9-cis retinoic acid
1159555	RAR_ant	retinoic acid receptor	antagonist	luciferase	C3RL4	Retinol	ER50891
1224893	CAR_ant	constitutive androstane receptor	antagonist	luciferase	HepG2	CITCO	PK11195
1224895	TSHR_ago	thyroid stimulating hormone receptor	agonist	cAMP assay	HEK293	Ro20-1724	thyroid stimulating hormone
1259247	ARfull2_ant	androgen receptor	antagonist	luciferase	MDA-MB	R1881	Nilutamide
1259248	ERfull_estra_ant	estrogen receptor alpha	antagonist	luciferase	BG1	17beta-estradiol	4-Hydroxy tamoxifen
1259387	ARant_ago	androgen receptor	agonist	luciferase	MDA-MB	Nilutamide	R1881
1259391	ERaant_ago	estrogen receptor alpha	agonist	luciferase	BG1	ICI-182,780	17beta-Estradiol
1259393	TSHR2_ago	thyroid stimulating hormone receptor	agonist	cAMP assay	HEK293	Ro20-1724	thyroid stimulating hormone
1259394	ERb_ago	estrogen receptor beta	agonist	beta-lactamase	HEK293T		17beta-Estradiol
1259395	TSHR_ant	thyroid stimulating hormone receptor	antagonist	cAMP assay	HEK293	thyroid stimulating hormone	Ro20-1724
1259396	ERb2_ant	estrogen receptor beta	antagonist	beta-lactamase	HEK293T	17beta-Estradiol	4-Hydroxy tamoxifen
1259403	ERR_ant	estrogen related receptor	antagonist	luciferase	HEK293		XTC790
1259404	ERR_ago	estrogen related receptor	agonist	luciferase	HEK293		Genistein
1347033	PXR_ago	pregnane X receptor	agonist	luciferase	HepG2		Rifampicin
1347036	PR_ago	progesterone receptor	agonist	beta-lactamase	HEK293T		R5020
1347038	TRHR_ant	thyrotropin-releasing hormone receptor	antagonist	intracellular calcium assay	HEK293	thyrotropin-releasing hormone	midazolam

NA: not analyzed.

## Supplementary Materials

The following are available online at https://www.mdpi.com/1420-3049/25/12/2764/s1, Figure S1. DeepSnap-DL procedure, Figure S2. Schematic illustrating how preparation of Tra/Val/Test datasets. Figure S3. Schematic view of GoogeLeNet archirecture. (a) Total layers used in this study. (b) Inception model within the GoogLeNet. Figure S4a. Average Accuracy values in Val and Test datasets in models of 35 NRs agonist/antagonist by the DeepSnap-DL. N = 2. Figure S4b. Average F and BAC values in Test dataset in models of 35 NRs agonist/antagonist by the DeepSnap-DL. N = 2. Figure S5. Comparison of prediction performances among four in vitro assays. (a) Loss in the Val dataset, (b) accuracy in the Val dataset, (c) accuracy in the Test dataset. Table S1: NRs and chemical compounds used in this study.

## Abbreviations

Acc (Test)	accuracy in the test dataset
AhR	aryl hydrocarbon receptor
AOP	adverse outcome pathway
AR	androgen receptor
AUC	area under the curve
Acc (Val)	accuracy in the validation dataset
BAC	balanced accuracy
BSA	bisphenol A
CAR	constitutive androstane receptor
CNN	convolutional neural network
DBD	DNA-binding domain
DIGITS	deep learning GPU training system
DL	deep learning
DMSO	dimethyl sulfoxide
ER	estrogen receptor
ERR	estrogen-related receptor
F	F-measure
FN	false negative
FP	false positive
FXR	farnesoid X receptor
GR	glucocorticoid receptor
LBD	ligand-binding domain
Loss (Val)	loss in the validation dataset
LXR	liver X receptor
MCC	Matthews correlation coefficient
ML	machine learning
MOE	molecular operating environment
NR	nuclear receptor
PPAR	peroxisome proliferator-activated receptor
PR	progesterone receptor
PXR	pregane X receptor
qHTS	quantitative high-throughput screening
QSAR	quantitative structure**–**activity relationship
RAR	retinoic acid receptor
RE	response element
RXR	retinoid X receptor
ROC	receiver operating characteristic
SE	standard error
SMILES	simplified molecular input line entry system
SSRM	selective steroid receptor modulator
TN	true negative
Tox21	Toxicology in the 21st Century
TP	true positive
TR	thyroid receptor
TSHR	thyroid-stimulating hormone receptor
VDR	vitamin D receptor
