# Supplementary material for: Molecular Image-Based Prediction Models of Nuclear Receptor Agonists and Antagonists Using the DeepSnap-Deep Learning Approach with the Tox21 10K Library
Source: Molecules. 2020 Jun 15;25(12):2764. doi: 10.3390/molecules25122764 (PMC7356846; doi:10.3390/molecules25122764)
Supplement: Supplementary file 1 [file molecules-25-02764-s001.pdf]

Figure 1

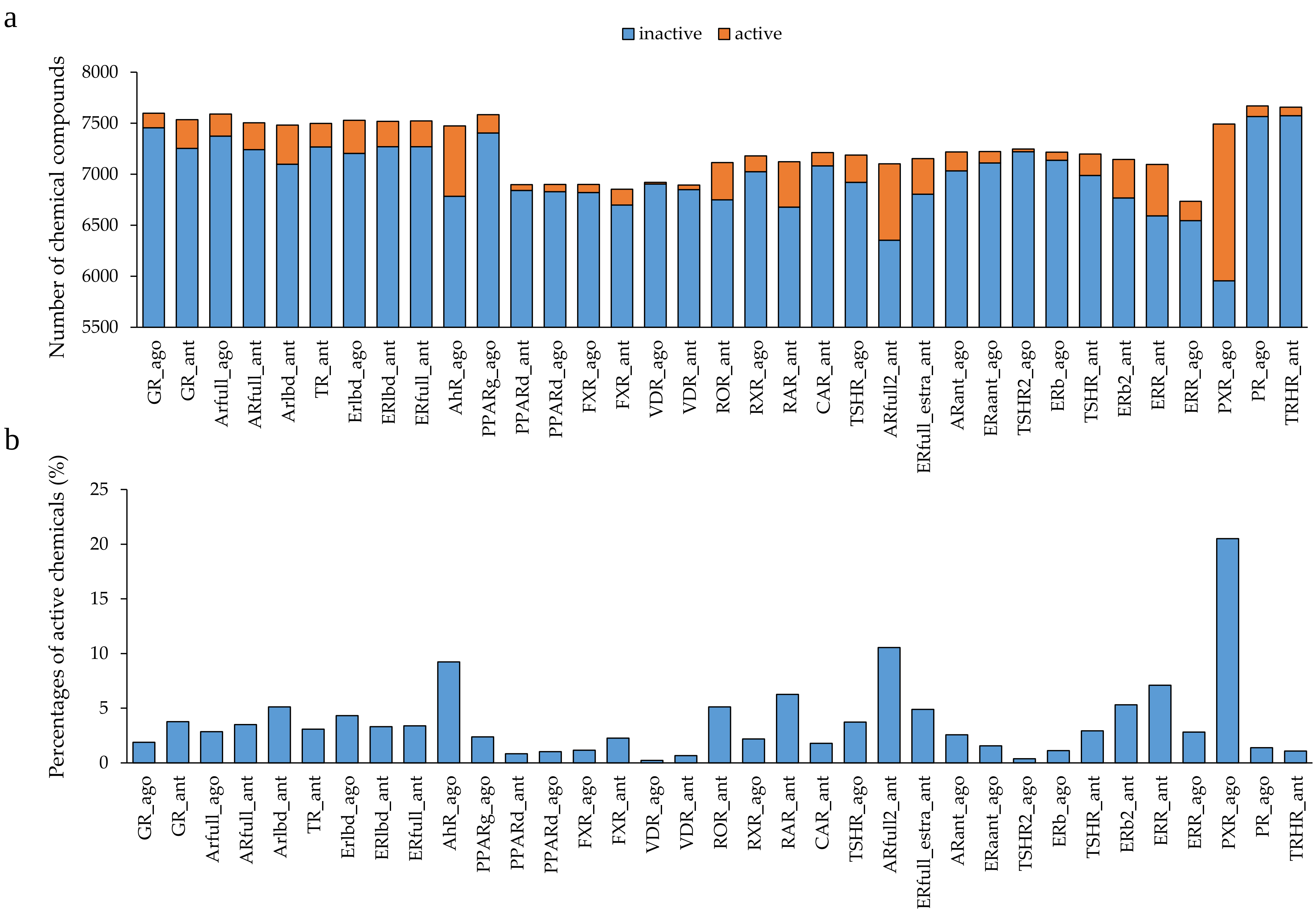
a
inactive
active
Number of chemical compounds
8000
7500
7000
6500
6000
5500
GR_ago
GR_ant
Arfull_ago
ARfull_ant
Arlbd_ant
TR_ant
Erlbd_ago
ERlbd_ant
ERfull_ant
AhR_ago
PPARg_ago
PPARd_ant
PPARd_ago
FXR_ago
FXR_ant
VDR_ago
VDR_ant
ROR_ant
RXR_ago
RAR_ant
CAR_ant
TSHR_ago
ARfull2_ant
ERfull_estra_ant
ARant_ago
ERaant_ago
TSHR2_ago
ERb_ago
TSHR_ant
ERb2_ant
ERR_ant
ERR_ago
PXR_ago
PR_ago
TRHR_ant
b
Percentages of active chemicals (%)
25
20
15
10
5
0
GR_ago
GR_ant
Arfull_ago
ARfull_ant
Arlbd_ant
TR_ant
Erlbd_ago
ERlbd_ant
ERfull_ant
AhR_ago
PPARg_ago
PPARd_ant
PPARd_ago
FXR_ago
FXR_ant
VDR_ago
VDR_ant
ROR_ant
RXR_ago
RAR_ant
CAR_ant
TSHR_ago
ARfull2_ant
ERfull_estra_ant
ARant_ago
ERaant_ago
TSHR2_ago
ERb_ago
TSHR_ant
ERb2_ant
ERR_ant
ERR_ago
PXR_ago
PR_ago
TRHR_ant

Supplementary Figure 1

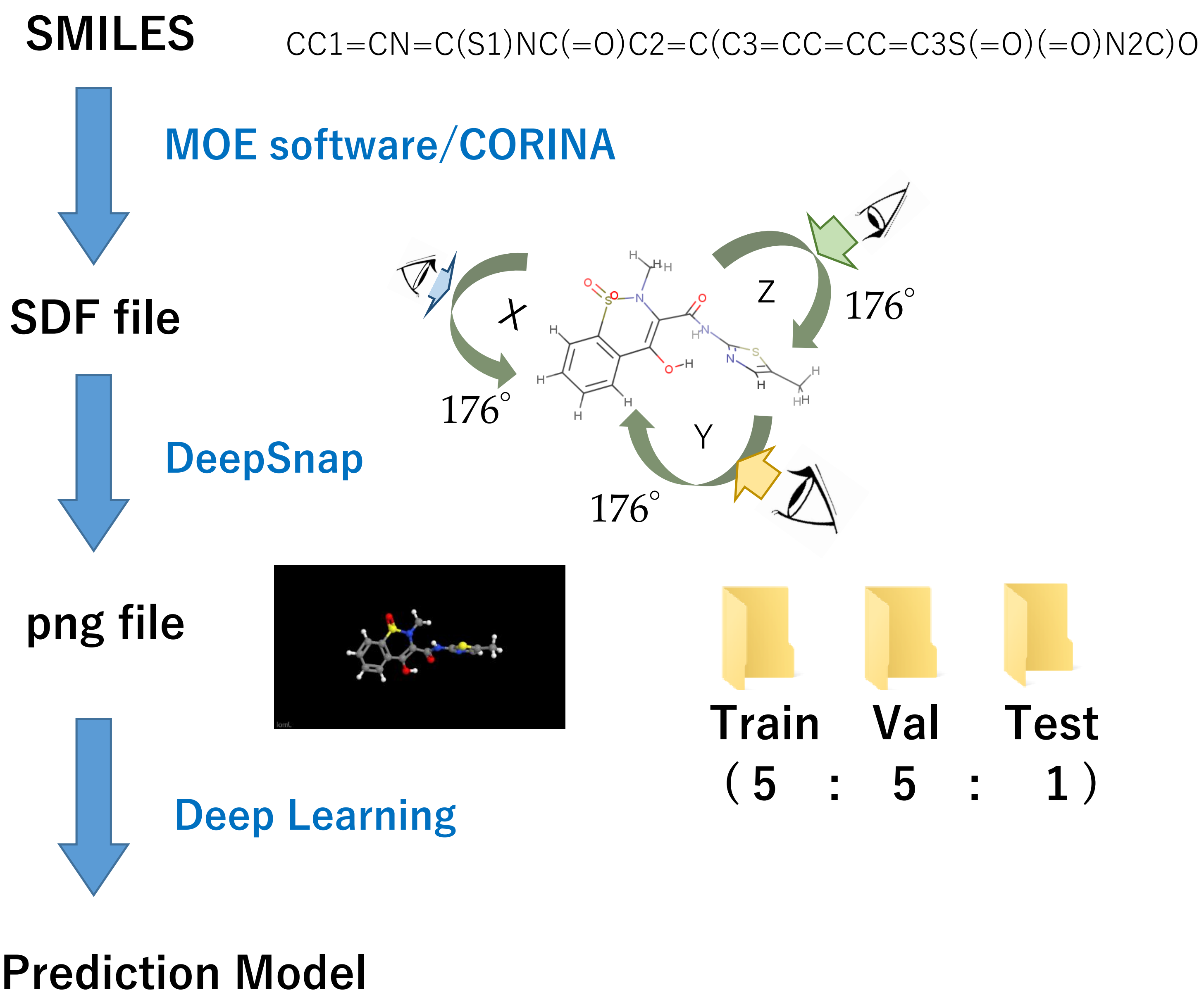

Figure 2

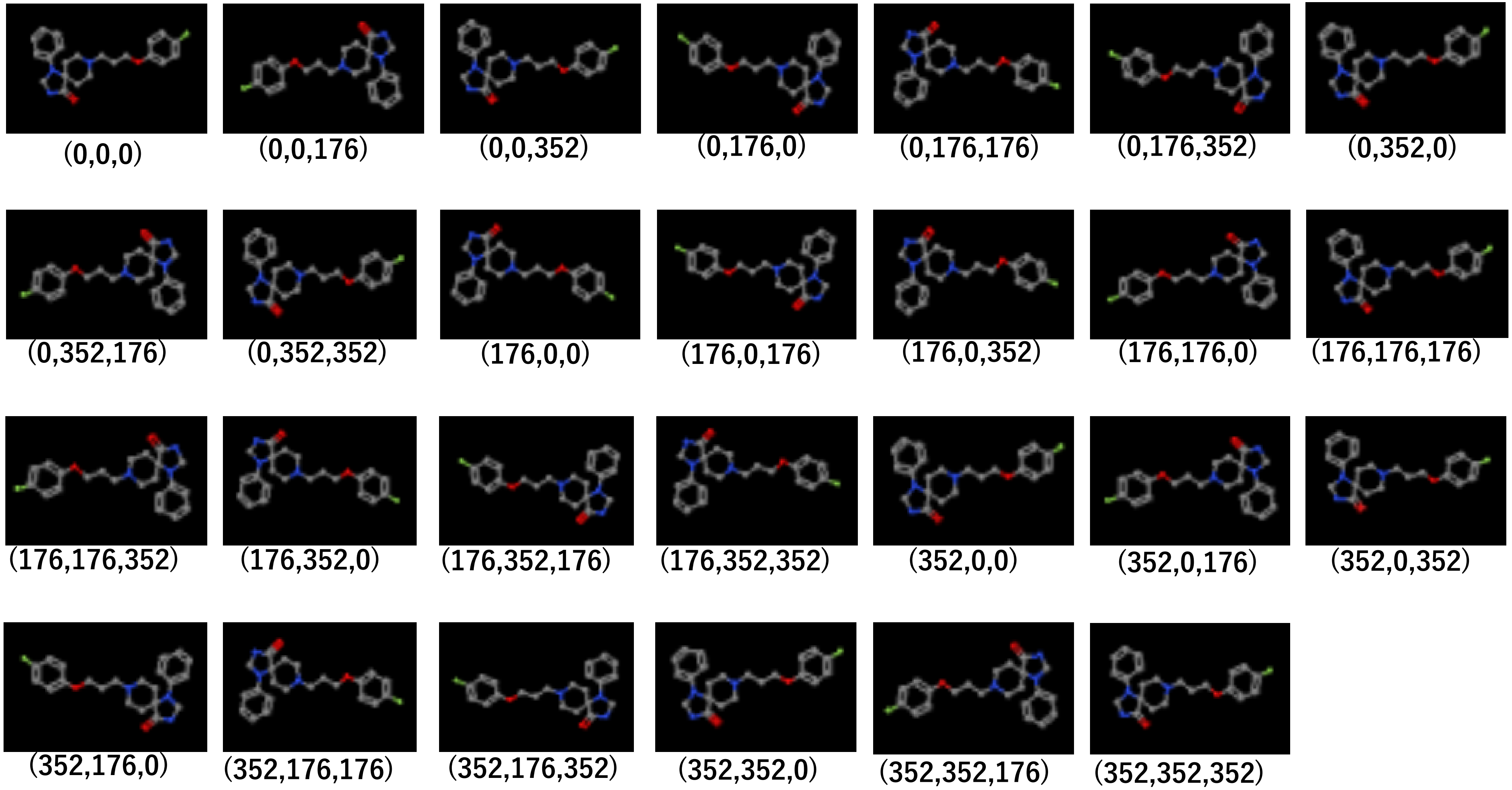
(0,0,0)
(0,0,176)
(0,0,352)
(0,176,0)
(0,176,176)
(0,176,352)
(0,352,0)
(0,352,176)
(0,352,352)
(176,0,0)
(176,0,176)
(176,0,352)
(176,176,0)
(176,176,176)
(176,176,352)
(176,352,0)
(176,352,176)
(176,352,352)
(352,0,0)
(352,0,176)
(352,0,352)
(352,176,0)
(352,176,176)
(352,176,352)
(352,352,0)
(352,352,176)
(352,352,352)

Supplementary Figure 2

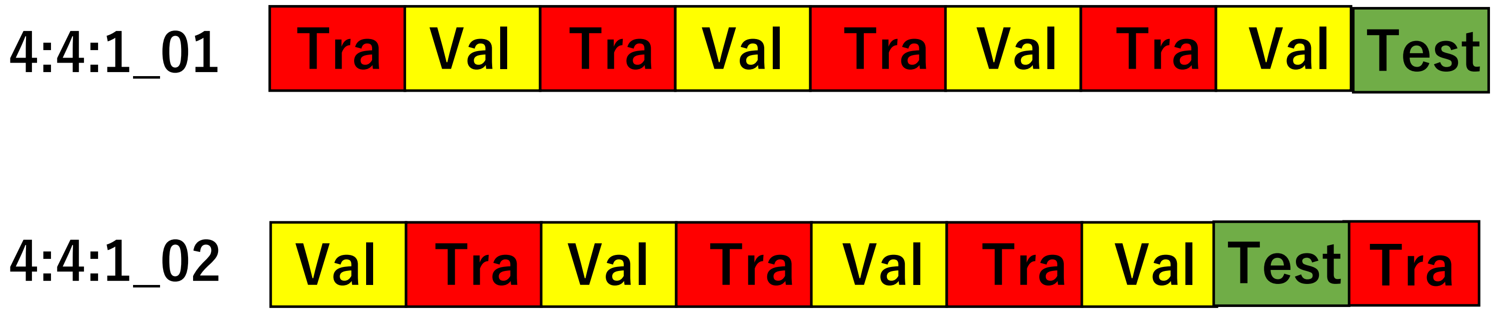

Supplementary Figure 3

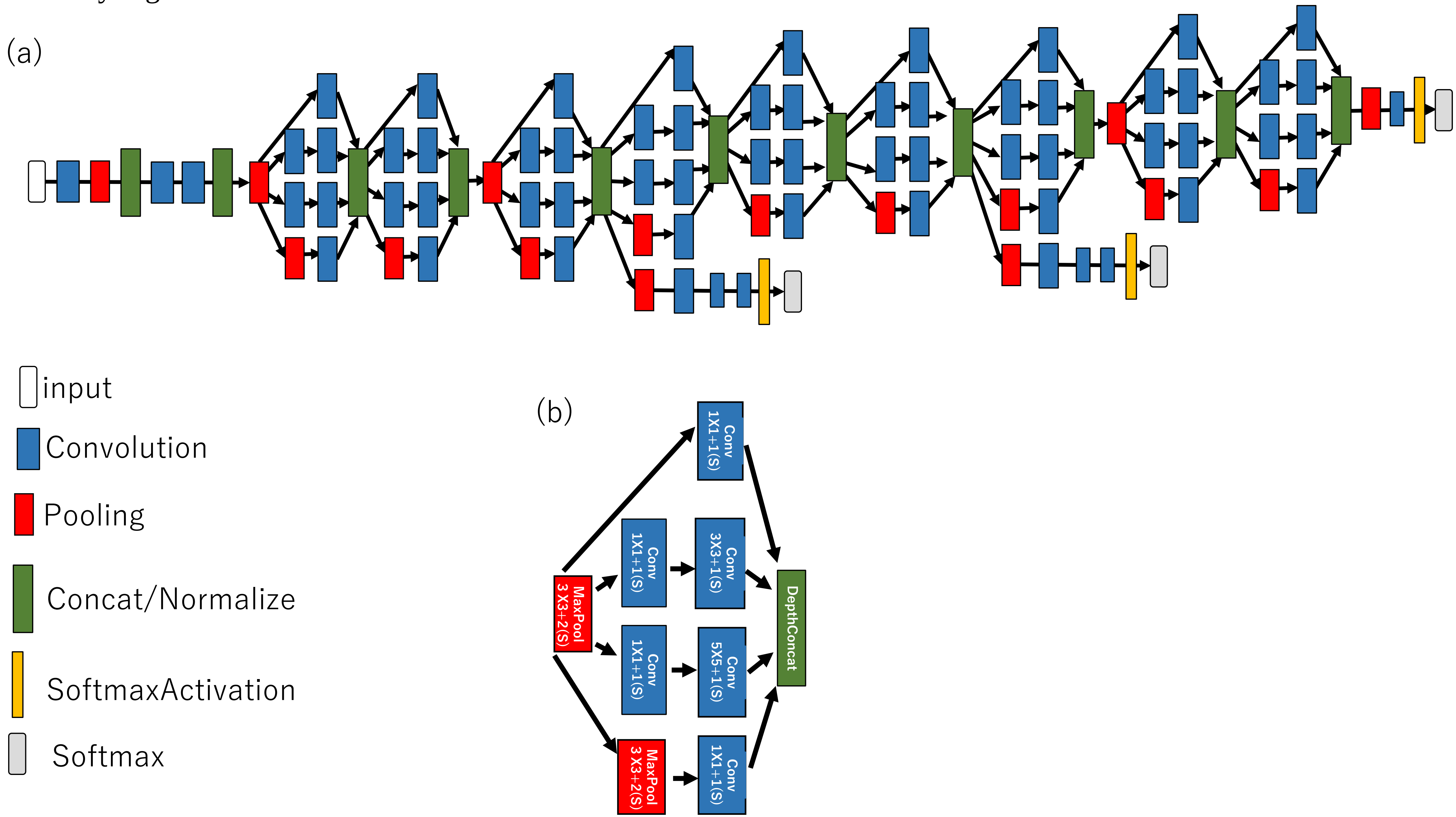

Figure 3

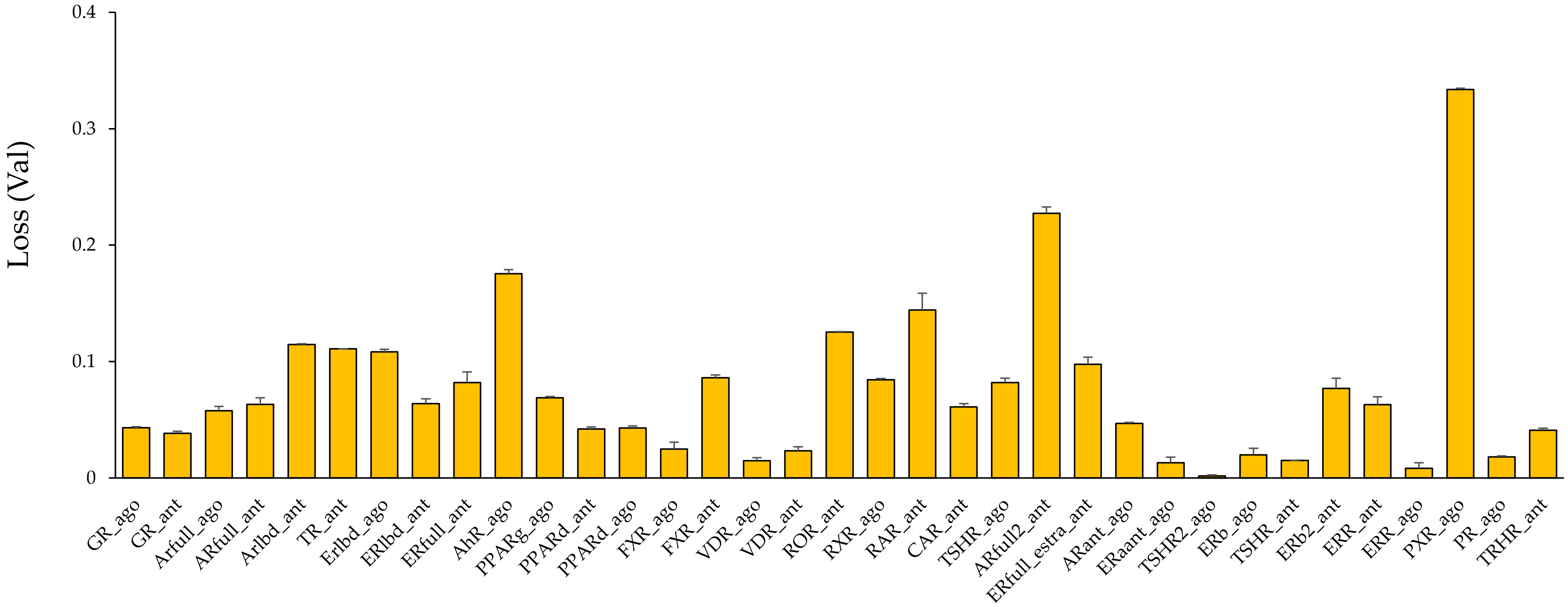

Loss (Val)
0.4
0.3
0.2
0.1
0
GR_ago
GR_ant
Arfull_ago
ARfull_ant
Arlbd_ant
TR_ant
Erlbd_ago
ERlbd_ant
ERfull_ant
AhR_ago
PPARg_ago
PPARd_ant
PPARd_ago
FXR_ago
FXR_ant
VDR_ago
VDR_ant
ROR_ant
RXR_ago
RAR_ant
CAR_ant
TSHR_ago
ARfull2_ant
ERfull_estra_ant
ARant_ago
ERaant_ago
TSHR2_ago
ERb_ago
TSHR_ant
ERb2_ant
ERR_ant
ERR_ago
PXR_ago
PR_ago
TRHR_ant

Supplementary Figure 4a

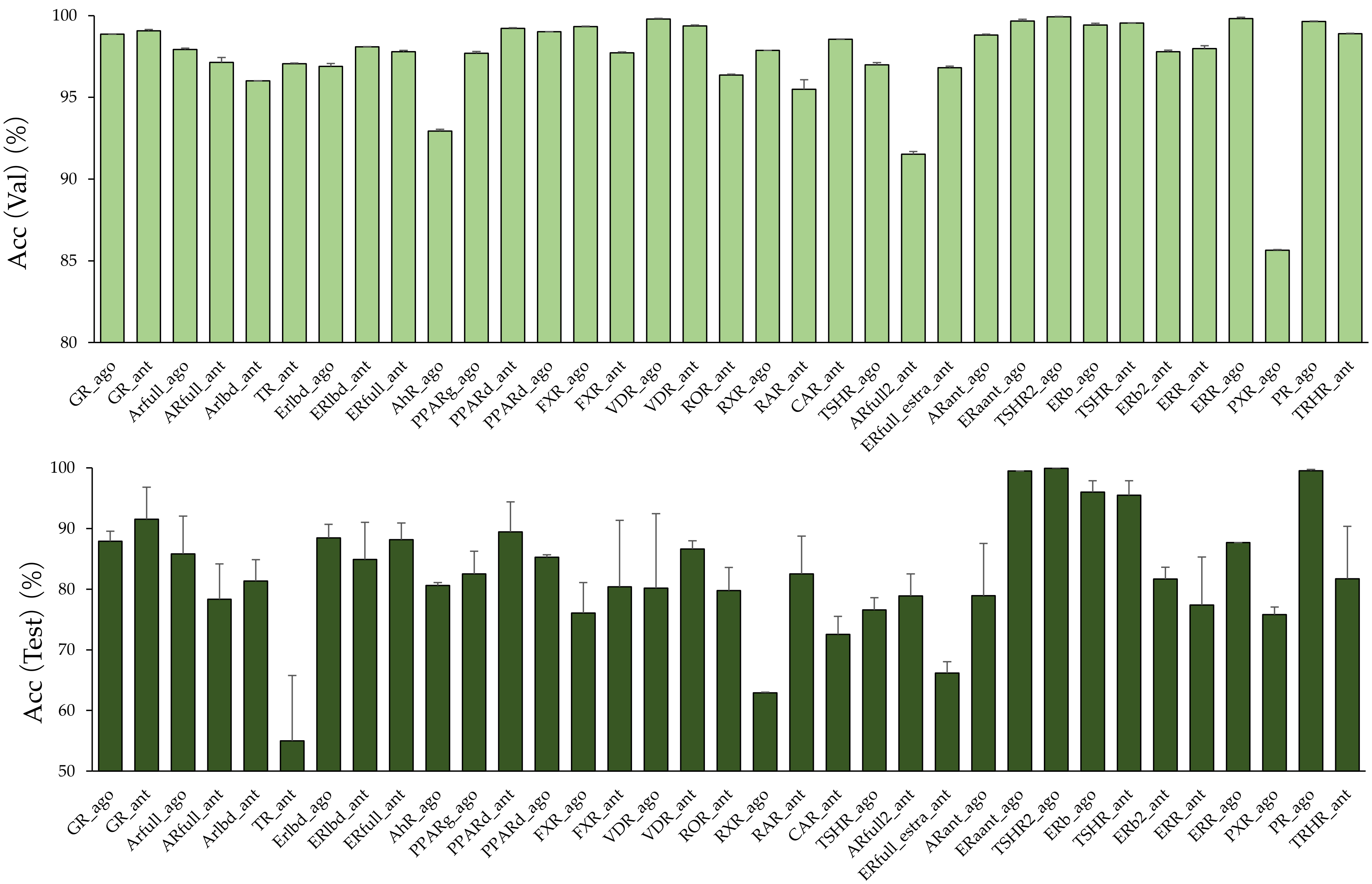

Figure 4

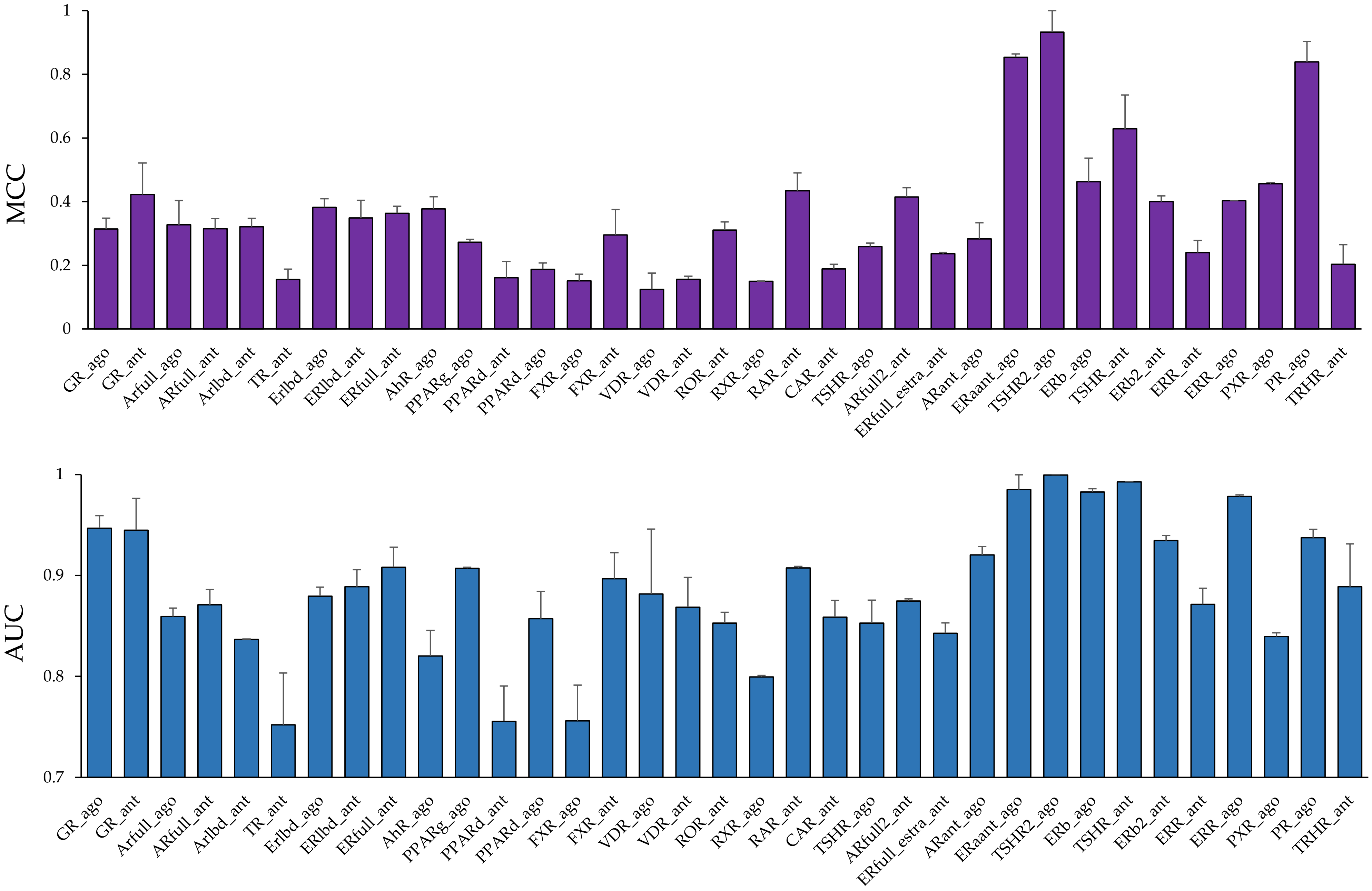

MCC
1
0.8
0.6
0.4
0.2
0
GR_ago
GR_ant
Arfull_ago
ARfull_ant
Arlbd_ant
TR_ant
Erlbd_ago
ERlbd_ant
ERfull_ant
AhR_ago
PPARg_ago
PPARd_ant
PPARd_ago
FXR_ago
FXR_ant
VDR_ago
VDR_ant
ROR_ant
RXR_ago
RAR_ant
CAR_ant
TSHR_ago
ARfull2_ant
ERfull_estra_ant
ARant_ago
ERaant_ago
TSHR2_ago
ERb_ago
TSHR_ant
ERb2_ant
ERR_ant
ERR_ago
PXR_ago
PR_ago
TRHR_ant
AUC
1
0.9
0.8
0.7

Supplementary Figure 4b

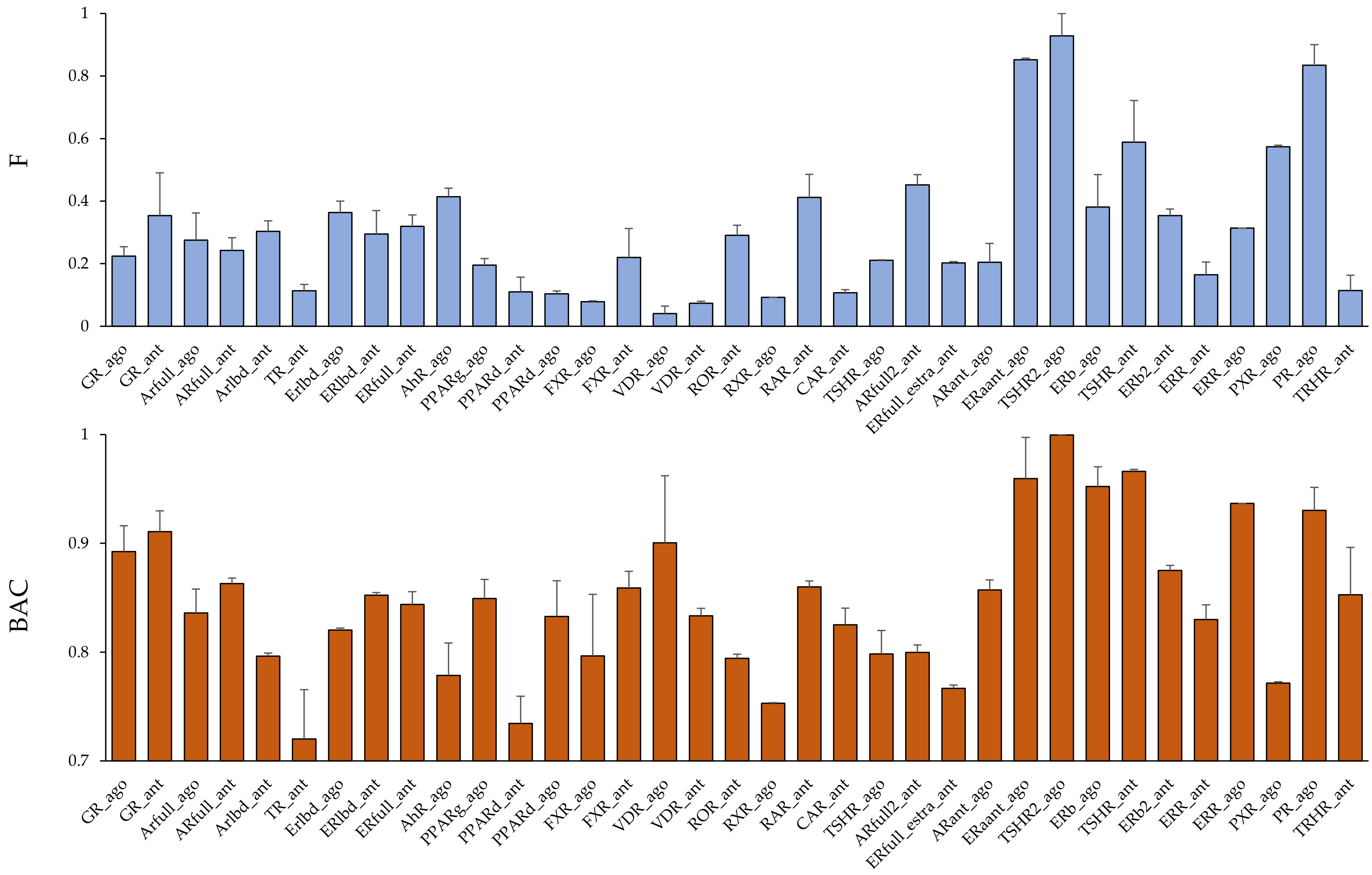

Figure 5

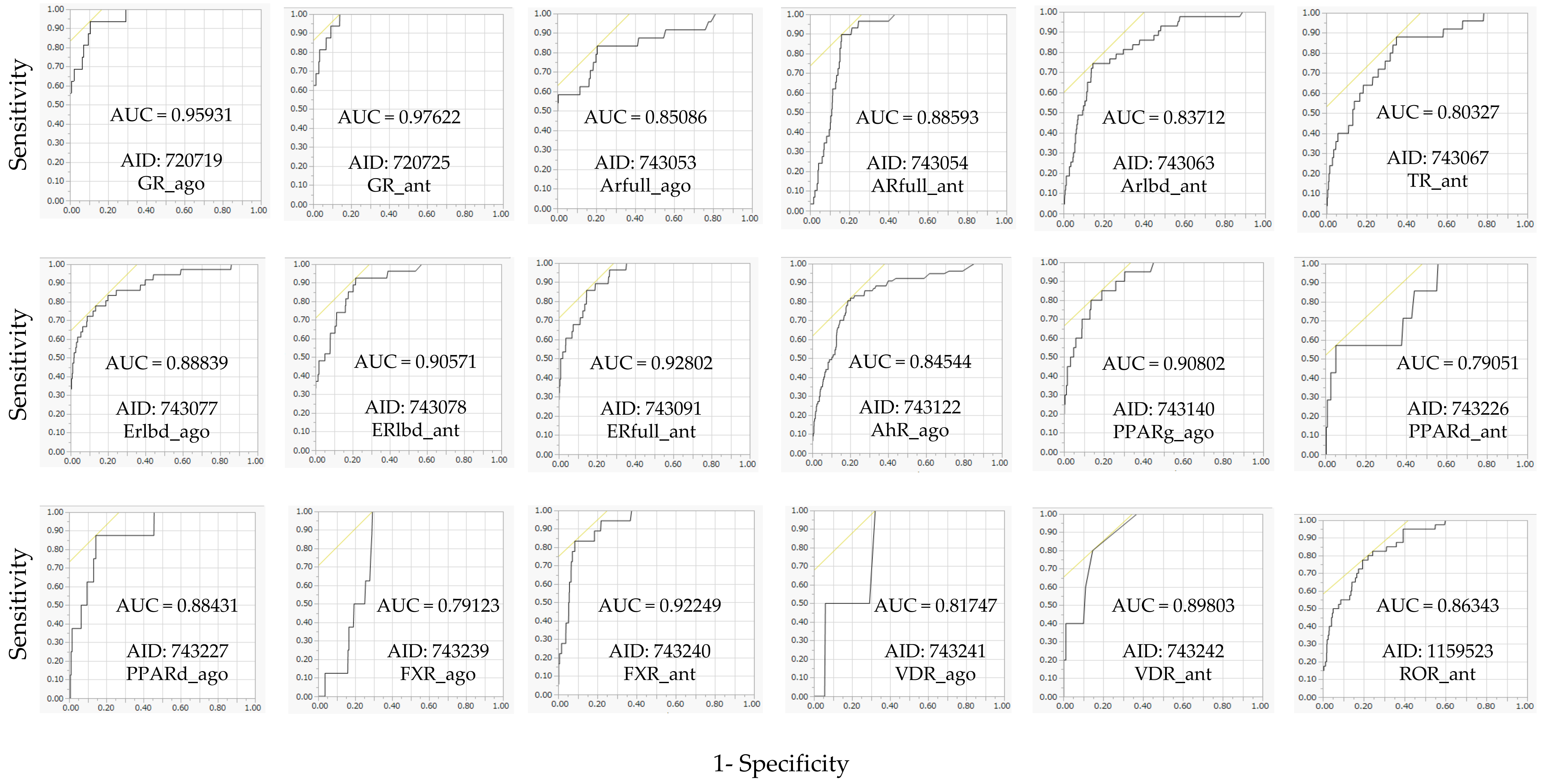

Sensitivity
AUC = 0.95931
AID: 720719
GR_ago
AUC = 0.97622
AID: 720725
GR_ant
AUC = 0.85086
AID: 743053
Arfull_ago
AUC = 0.88593
AID: 743054
ARfull_ant
AUC = 0.83712
AID: 743063
Arlbd_ant
AUC = 0.80327
AID: 743067
TR_ant
Sensitivity
AUC = 0.88839
AID: 743077
Erlbd_ago
AUC = 0.90571
AID: 743078
ERlbd_ant
AUC = 0.92802
AID: 743091
ERfull_ant
AUC = 0.84544
AID: 743122
AhR_ago
AUC = 0.90802
AID: 743140
PPARg_ago
AUC = 0.79051
AID: 743226
PPARd_ant
Sensitivity
AUC = 0.88431
AID: 743227
PPARd_ago
AUC = 0.79123
AID: 743239
FXR_ago
AUC = 0.92249
AID: 743240
FXR_ant
AUC = 0.81747
AID: 743241
VDR_ago
AUC = 0.89803
AID: 743242
VDR_ant
AUC = 0.86343
AID: 1159523
ROR_ant
1- Specificity

Figure 5

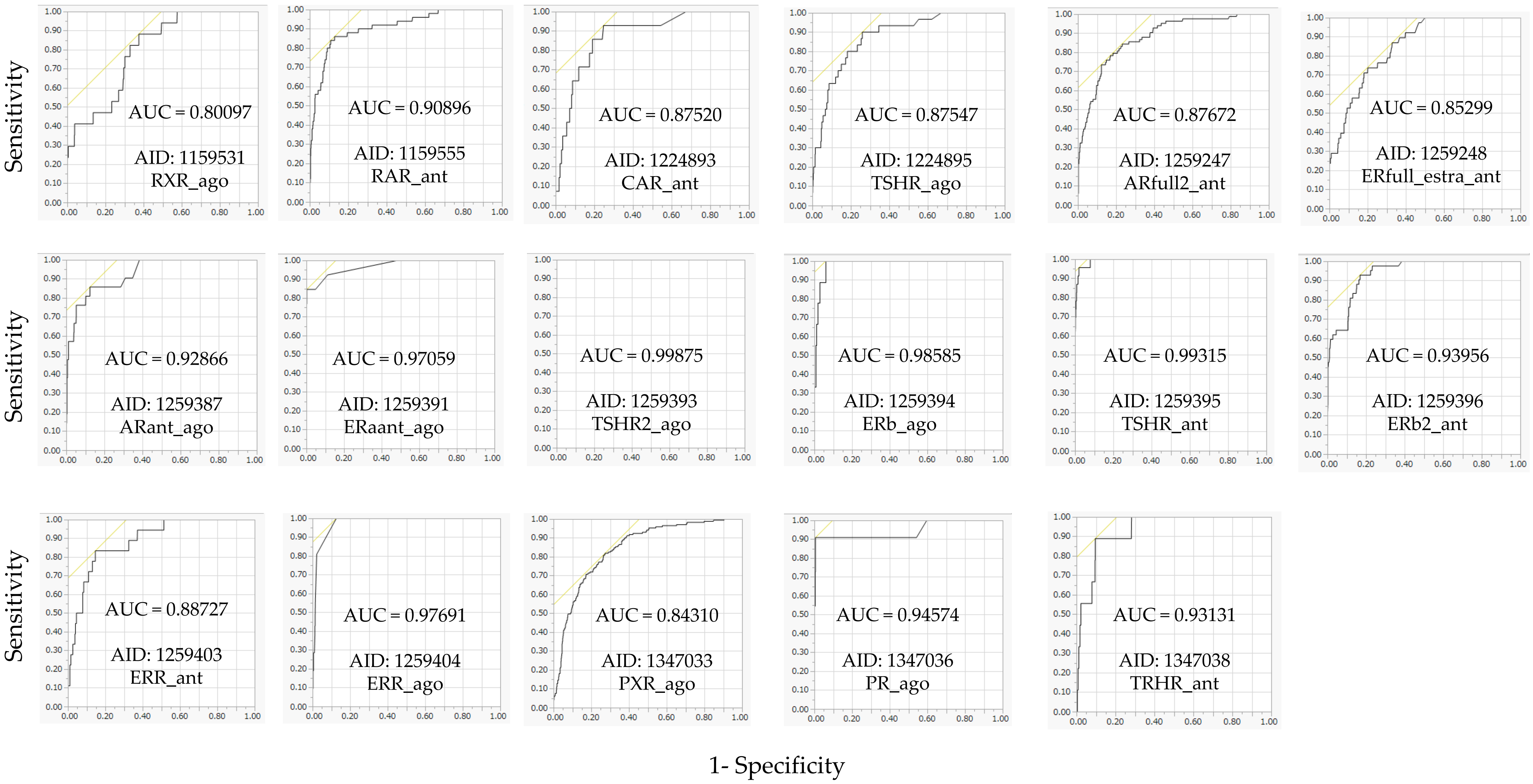
Sensitivity
AUC = 0.80097
AID: 1159531
RXR_ago
AUC = 0.90896
AID: 1159555
RAR_ant
AUC = 0.87520
AID: 1224893
CAR_ant
AUC = 0.87547
AID: 1224895
TSHR_ago
AUC = 0.87672
AID: 1259247
ARfull2_ant
AUC = 0.85299
AID: 1259248
ERfull_estra_ant
AUC = 0.92866
AID: 1259387
ARant_ago
AUC = 0.97059
AID: 1259391
ERaant_ago
AUC = 0.99875
AID: 1259393
TSHR2_ago
AUC = 0.98585
AID: 1259394
ERb_ago
AUC = 0.99315
AID: 1259395
TSHR_ant
AUC = 0.93956
AID: 1259396
ERb2_ant
AUC = 0.88727
AID: 1259403
ERR_ant
AUC = 0.97691
AID: 1259404
ERR_ago
AUC = 0.84310
AID: 1347033
PXR_ago
AUC = 0.94574
AID: 1347036
PR_ago
AUC = 0.93131
AID: 1347038
TRHR_ant
1- Specificity

Figure 6

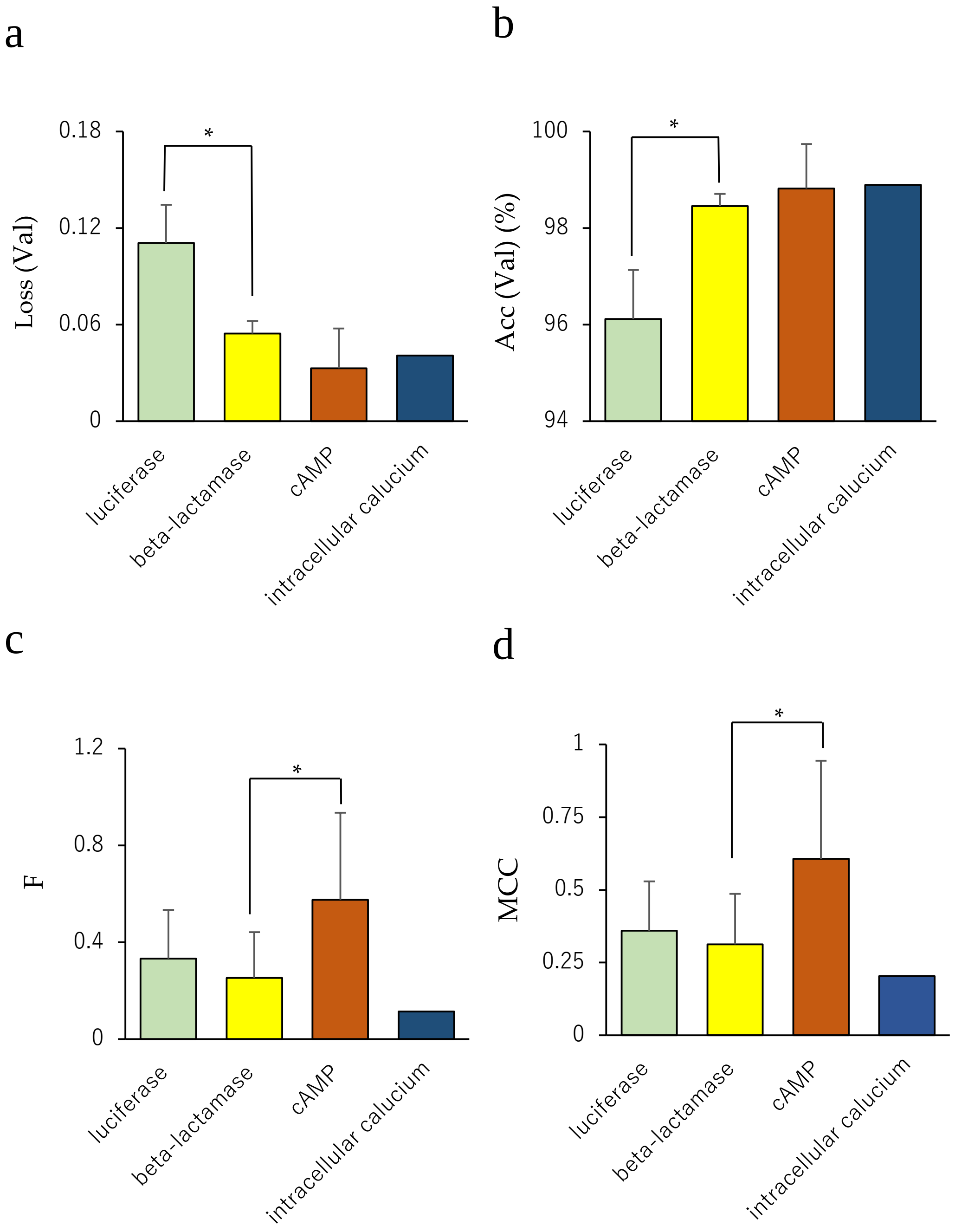
a
b
c
d
Loss (Val)
0.18
0.12
0.06
0
*
Acc (Val) (%)
100
98
96
94
F
1.2
0.8
0.4
MCC
1
0.75
0.5
0.25
luciferase
beta-lactamase
cAMP
intracellular calucium

Supplementary Figure 5

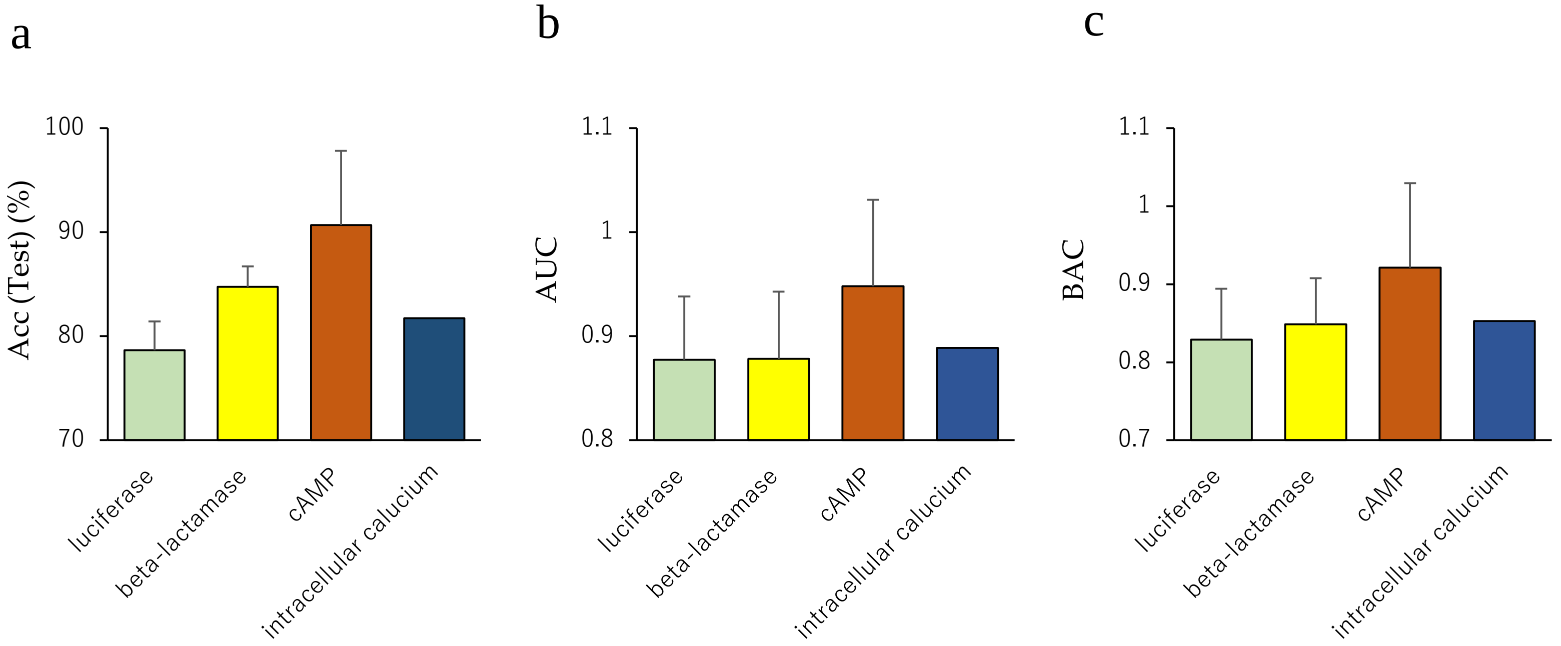

Table S1. Nuclear receptors and chemical compounds used in this study.

		tra		val		test		total	
PubChem AID	model names	active	inactive	active	inactive	active	inactive	active	inactive
720719	GR_ago	64	3313	63	3314	16	828	143	7455
720725	GR_ant	126	3224	126	3223	31	806	283	7253
743053	Arfull_ago	96	3278	96	3277	24	819	216	7374
743054	ARfull_ant	117	3218	117	3218	29	805	263	7241
743063	Arlbd_ant	171	3155	170	3155	42	789	383	7099
743067	TR_ant	103	3230	102	3230	26	807	231	7267
743077	Erlbd_ago	145	3202	144	3202	36	801	325	7205
743078	ERlbd_ant	111	3231	111	3231	27	808	249	7270
743091	ERfull_ant	113	3231	113	3231	28	808	254	7270
743122	AhR_ago	307	3015	307	3015	76	754	690	6784
743140	PPARg_ago	80	3291	80	3291	20	823	180	7405
743226	PPARd_ant	25	3041	25	3041	7	760	57	6842
743227	PPARd_ago	31	3036	32	3035	8	759	71	6830
743239	FXR_ago	35	3032	36	3031	9	758	80	6821
743240	FXR_ant	69	2977	69	2977	17	744	155	6698
743241	VDR_ago	7	3069	6	3069	2	767	15	6905
743242	VDR_ant	20	3045	20	3044	5	761	45	6850
1159523	ROR_ant	162	3000	162	3000	40	750	364	6750
1159531	RXR_ago	70	3122	69	3122	17	781	156	7025
1159555	RAR_ant	198	2968	198	2968	50	742	446	6678
1224893	CAR_ant	58	3148	57	3148	14	787	129	7083
1224895	TSHR_ago	119	3076	119	3076	30	769	268	6921
1259247	ARfull2_ant	333	2824	333	2823	83	706	749	6353
1259248	ERfull_estra_ant	155	3025	155	3024	39	756	349	6805
1259387	ARant_ago	82	3126	82	3126	21	781	185	7033
1259391	ERaant_ago	50	3160	50	3160	12	790	112	7110
1259393	TSHR2_ago	12	3210	12	3209	3	802	27	7221
1259394	ERb_ago	36	3172	35	3172	9	793	80	7137
1259395	TSHR_ant	93	3106	93	3106	24	776	210	6988
1259396	ERb2_ant	168	3008	169	3007	42	752	379	6767
1259403	ERR_ant	224	2930	224	2930	56	733	504	6593
1259404	ERR_ago	84	2910	84	2909	21	727	189	6546
1347033	PXR_ago	683	2647	684	2646	170	662	1537	5955
1347036	PR_ago	48	3362	47	3362	11	841	106	7565
1347038	TRHR_ant	37	3366	37	3366	9	842	83	7574
